# Health system readiness—the neglected catalyst in Malaria elimination

**DOI:** 10.1186/s12936-026-06059-9

**Published:** 2026-07-23

**Authors:** Win Htike, Win Han Oo, Catherine M. Bennett, Paul A. Agius, Alyssa E. Barry, Freya J. I. Fowkes

**Affiliations:** 1 Disease Elimination Programme, Burnet Institute, Melbourne, VIC Australia; 2 Health Security and Malaria Programme, Burnet Institute Myanmar, Yangon, Myanmar; 3 Institute for Mental and Physical Health and Clinical Translation, School of Medicine, Deakin University, Waurn Ponds, VIC Australia; 4 Centre for Epidemiology and Biostatistics, Melbourne School of Population and Global Health, University of Melbourne, Melbourne, VIC Australia; 5 Institute for Health Transformation, Deakin University, Waurn Ponds, VIC Australia; 6 Biostatistics Unit, Faculty of Health, Deakin University, Waurn Ponds, VIC Australia; 7 Department of Epidemiology and Preventive Medicine, Monash University, Melbourne, VIC Australia

**Keywords:** Assessment, Health systems, Malaria elimination, Readiness

## Abstract

**Background:**

While strong political commitment and robust technical strategies are essential to drive malaria elimination, the readiness of national health systems ultimately determines whether these commitments can be successfully translated into action.

**Main text:**

Without adequate preparation, gaps in the health system can undermine the transition from malaria burden reduction to elimination policies, reduce the effectiveness of interventions, and jeopardise progress toward achieving zero local transmission of malaria. Despite its critical importance, there is currently no systematic and comprehensive approach for assessing health system readiness for malaria elimination. This paper highlights the consequences of unprepared health systems and the urgent need for standardised, structured, comprehensive readiness assessments that would enable policymakers and programme managers to systematically evaluate health system readiness for malaria elimination at national and subnational levels, identify and address critical gaps, and strengthen the health systems. These efforts will support sustained elimination and achieve WHO-certified malaria elimination.

## Background

In 2015, the Sixty-Eighth World Health Assembly urged its 91 malaria-endemic Member States to accelerate their progress towards malaria elimination (zero incidence of indigenous cases) and prevent re-establishment of malaria [1]. Since then, 17 countries have been certified as “malaria-free” (three consecutive years of zero indigenous cases) by the World Health Organization (WHO) [2]. Others have either requested certification (e.g., Türkiye), reported zero indigenous cases for at least two consecutive years (e.g., Bhutan, Malaysia, Saudi Arabia), or committed to eliminating malaria by 2030 through regional initiatives such as the Southern Africa Elimination 8 (E8) Initiative (Angola, Botswana, Eswatini, Mozambique, Namibia, South Africa, Zambia, and Zimbabwe) and the Mekong Malaria Elimination programme (Cambodia, Lao People’s Democratic Republic, Myanmar, Thailand, and Viet Nam) [2, 3].

To obtain WHO certification, national malaria programmes must undergo a highly scrutinised review to confirm nationwide interruption of transmission [4]. Meeting these standards requires a well-prepared health system from the outset of elimination strategy implementation. Premature reorientation of malaria programmes from control to elimination, without ensuring adequate health system capacity—including technical capability, robust surveillance, a trained workforce, and sustained financing—can jeopardise elimination progress. However, despite its central importance, systematic assessment of health system readiness remains neglected in the global malaria elimination discourse. This perspective highlights the implications of unprepared health systems and calls for greater prioritisation of health system readiness assessments to guide the transition from malaria control to elimination strategies.

### Critical role of health system readiness in moving towards malaria elimination

To eliminate malaria, health systems must be adequately prepared to deliver the WHO Global Technical Strategy for Malaria. The strategy is built around three core pillars: ensuring universal access to malaria prevention, diagnosis, and treatment services; accelerating efforts towards elimination; and transforming malaria surveillance into a key intervention [5]. When a country reorients its malaria programme from control to elimination, existing testing, treatment, and vector control services must be enhanced and optimised to ensure universal access (Table 1). Additionally, the country must accelerate its elimination efforts by securing strong political commitment and support, sustaining programme financing, and implementing population-wide parasite clearance and targeted interventions such as reactive indoor residual spraying, transmission-blocking chemotherapy, and mass drug administration [5].

**Table 1:** Key distinctions between malaria control and elimination phases, with corresponding health system needs for transitioning to and sustaining elimination

Key activities during control phase	Key activities during elimination phase	Health system needs for transition to and maintenance of elimination	
Pillar 1: universal access to malaria services			
Early diagnosis and appropriate treatment Vector control and personal protection	Enhanced and optimised case management to ensure universal access Enhanced and optimised vector control and personal protection to ensure universal access	Service delivery:	Adequate healthcare facilities and service providers to ensure universal access
		Surveillance:	Good documentation practices
		Workforce:	Adequate number of properly trained staff
		Commodities:	Uninterrupted supply of essential items (e.g., rapid diagnostic tests, medicines)
		Financing:	Sufficient funding for delivering malaria services
		Governance:	Mandatory reporting of malaria cases
			Strong technical leadership from the national programme Strong partnership and coordination
Pillar 2: accelerating efforts towards elimination			
Not applicable	Strong political commitment and support Sustained programme financing for the last-mile of elimination Population-wide parasite clearance and/or targeted interventions such as indoor residual spraying, transmission-blocking chemotherapy, and mass drug administration	Service delivery:	Universal coverage of malaria services
		Surveillance:	Strong surveillance systems on vector, drug resistance, and insecticide resistance
		Workforce:	Strong technical expertise and experience
		Commodities:	Uninterrupted supply of essential items (e.g., chemical for indoor residual spraying, long-lasting insecticidal treated nets)
		Financing:	Sustainable funding support
		Governance:	High-level commitment and coordination
Pillar 3: transforming malaria surveillance into a key intervention			
Routine reporting of malaria cases	Case-based reactive surveillance and response (e.g. case notification, case and foci investigation and response) to detect, characterise, respond and monitor all cases and foci	Service delivery:	Prompt diagnosis, treatment, and notification
		Surveillance:	Timely implementation of response activities
		Workforce:	Adequate number of properly trained staff
		Commodities:	Uninterrupted supply of essential equipment (e.g., reporting forms, surveillance manuals and guidelines)
		Financing:	Sufficient funding for response activities
		Governance:	Mandatory reporting
			Standardised guidelines and standard operating procedures

Adapted from the WHO Global Technical Strategy for Malaria [5]

Furthermore, malaria surveillance systems must be strengthened and repositioned as key interventions to detect, characterise, respond to, and monitor all malaria cases and transmission foci [5]. While political commitment and technical strategies are required to galvanise and guide malaria elimination, translating this commitment into action depends on countries’ health system readiness. This readiness is underpinned by six interdependent WHO health system building blocks—service delivery, surveillance, workforce, commodities, financing, and governance—that must operate as a coherent system [6]. Gaps in any one component can create bottlenecks that limit overall system performance and undermine implementation across the three pillars.

Drawing on lessons from countries that have achieved WHO malaria elimination certification, the effective implementation of optimised case management and vector control, targeted interventions, and strengthened surveillance requires a well-prepared health system, including service delivery, surveillance, workforce, commodities, financing and governance (Table 1) [7–10]. As countries accelerate progress towards malaria elimination, these health system building blocks must be able to support enhanced testing, treatment, vector control, and surveillance, aligned with the three pillars of the WHO Global Technical Strategy framework [11, 12].

A resilient and well-prepared health system is the foundation for successful malaria elimination and the prevention of re-establishment of transmission. As countries transition from malaria control to elimination, the same health system must accommodate newly integrated elimination strategies—such as timely detection of all infections and appropriate management of transmission foci to prevent onwards transmission—alongside existing services. This shift places additional demands on already stretched systems, making health system readiness a critical success factor. If underlying health system constraints are not identified and addressed before transitioning to an elimination agenda, they can be exacerbated, undermining the effectiveness of elimination efforts. Therefore, proactively strengthening the health system by identifying gaps in service delivery, surveillance, workforce, products, financing, and governance infrastructure is essential. Introducing malaria elimination strategies without a thorough assessment and preparation of the health system can lead to implementation challenges across all three pillars of the WHO Global Technical Strategy, resulting in disruptions to malaria prevention, diagnosis, and treatment services, hindered elimination efforts, and weakened surveillance [13–22].

### Unprepared health systems challenge malaria elimination

#### Pillar 1: universal access to malaria services

According to the WHO framework for malaria elimination, universal access to malaria prevention, diagnosis and treatment needs to be ensured during the elimination phase (Table 1) [23]. As transmission declines, malaria cases become concentrated among certain populations (“hot pops”, such as mobile and migrant populations), and in specific geographical areas (“hotspots”, such as border areas) [24]. Despite the importance of ensuring that malaria services are accessible to high-risk groups, achieving universal access for these populations is often constrained by health system limitations—such as limited access to healthcare services, workforce shortages, stock-outs, and insufficient funding—as well as external factors, including language barriers and the legal status of these groups [25].

Prevention, diagnosis, and treatment services—the main interventions for the control phase—are the cornerstone of elimination efforts [23]. However, countries face significant challenges in ensuring early diagnosis and appropriate treatment and providing vector control and personal protection services due to various health system issues, especially in resource-limited settings. Case studies from Zimbabwe and Tanzania reported frequent stock-outs of key medicines for prophylaxis (sulfadoxine-pyrimethamine) and treatment (artemisinin-based combination therapies), resulting in service disruptions that increased the risk of malaria resurgence and vulnerability among high-risk populations [13, 14]. Geographic isolation and inadequate healthcare infrastructure in hard-to-reach areas further delay the delivery of prevention, diagnosis, and treatment services—stagnating progress in malaria control efforts [26]—while weak partnerships and poor coordination between national programmes and relevant stakeholders also hinder the achievement of optimal coverage [15]. When health systems struggle to maintain these fundamental services, it is unlikely that they can adapt and scale up interventions to ensure universal access and fulfil the commitment to leave no one behind in elimination efforts.

Another example highlighting the importance of health system readiness is the use of quantitative point-of-care glucose-6-phosphate dehydrogenase (G6PD) tests in managing vivax malaria cases. *Plasmodium vivax*, the predominant malaria species in Asia and Latin America, poses a major challenge in elimination settings due to relapses caused by dormant liver-stage parasites [27], which require treatment with 8-aminoquinolines—drugs that may cause adverse effects in individuals with G6PD deficiency [28]. Studies in Brazil and Southeast Asia have demonstrated that point-of-care G6PD tests are user friendly, increase patient safety, promote treatment adherence, and contribute to radical cures of vivax malaria [29, 30]. Nonetheless, some national programmes in resource-limited settings introduced G6PD testing policies without assessing health system readiness, leading to barriers such as supply chain and storage issues, additional costs, and insufficient funding for training and quality control [31, 32]. These gaps have compromised the safe delivery of 8-aminoquinolines, reduced treatment adherence, and hindered radical cure [32]. Consequently, healthcare service providers could lose the community’s trust, which may lead to reduced patient motivation to access their services [33, 34]. This example illustrates how even a proven intervention can falter when deployed without readiness assessment and within under-resourced health systems.

#### Pillar 2: accelerating efforts towards elimination

As countries accelerate progress towards malaria elimination, efforts must prioritise areas with ongoing malaria transmission by implementing population-wide parasite clearance and targeted interventions such as indoor residual spraying, transmission-blocking chemotherapy, and mass drug administration [5]. The effectiveness and coverage of these activities largely depend on the health system’s capacity to deliver services to all at-risk populations [35]. Health systems must be supported by a workforce with strong technical expertise and experience, as well as an uninterrupted supply of essential items [29, 34]. Furthermore, to accelerate progress towards elimination and effectively implement the strategies outlined in the WHO Global Technical Strategy for Malaria (Table 1) [5], national programmes must secure adequate and sustainable funding [36].

Interrupting onwards malaria transmission is more expensive than reducing disease burden. This is because elimination programmes typically require greater investment to optimise service coverage in hotspot areas—which are often hard and costly to reach—implement targeted interventions to interrupt transmission, and reorient programmes to strengthen surveillance systems [37]. Sustained and adequate funding is therefore essential, as disruptions to elimination activities can lead to a resurgence of malaria cases [36]. Many low- and middle-income countries rely heavily on international funding for malaria elimination [38, 39]. Recently, major international donors, such as the United States President’s Malaria Initiative and the Global Fund, have either ceased or significantly reduced their malaria funding [40, 41], weakening the ability of the health system to implement malaria elimination strategies efficiently [42]. This decline in funding has disrupted service delivery, surveillance, workforce sustainability, and the supply of essential products and commodities [43]. Consequently, these countries must now prioritise and allocate limited resources strategically to strengthen health systems and advance malaria elimination.

The transition away from donor funding also risks undermining the sustainability of malaria programmes. Reduced external support may weaken political prioritisation and strain financing, governance, and coordination. Key programme components—particularly commodity supply chains, surveillance systems, and malaria-specific workforce capacity—are especially vulnerable to disruption [43]. Without careful transition planning, these gaps could lead to reduced intervention coverage and resurgence of transmission. Strengthening domestic financing, integrating malaria into broader health systems, and safeguarding essential functions will be critical to managing this transition.

Countries aiming to eliminate malaria often benefit from strong political commitment at the national level [44]. However, such commitment does not always translate into readiness at the subnational level. Gaps in health system capacity between the national and subnational levels impede accelerated efforts towards elimination by undermining the effectiveness of the service delivery and surveillance activities outlined in the WHO Global Technical Strategy (Table 1) [45]. For example, national programmes in resource-limited settings face service interruptions that challenge universal coverage, workforce shortages that limit technical expertise and experience, delays in reactive surveillance and response activities that weaken surveillance systems, and stock-outs of essential items that disrupt uninterrupted supply to populations at risk, all of which threaten the progress towards malaria elimination [16, 34]. It is therefore essential that both national and subnational health systems are adequately assessed and prepared to translate political commitment into effective action and accelerate efforts towards elimination.

#### Pillar 3: transforming malaria surveillance into a core intervention

In elimination settings, malaria surveillance must include timely detection of all infections, rapid and radical treatment to prevent onwards transmission, and thorough investigation and appropriate management of transmission foci (Table 1) [23]. These interconnected activities—collectively referred to as reactive surveillance and response activities—are critical to interrupt malaria transmission [46]. The extent to which malaria transmission could be interrupted largely depends on the timeliness and completeness of these time-bound reactive surveillance and response activities [47]. A notable example of a time-bound reactive surveillance and response strategy is the “1–3–7 approach” initiated by China in 2012 [48]. China successfully eliminated malaria by applying the “1–3–7 approach”, in which malaria cases were notified within 1 day and investigated within 3 days, and associated foci were investigated and responded to inhibit secondary transmission within 7 days [49]. The success of the “1–3–7 approach” in China was largely attributable to readiness in the health system, including continuous capacity building of health staff, robust information technology infrastructure, real-time reporting systems, and strong government commitment [10].

Following China’s success in malaria elimination, the “1–3–7 approach” has been adopted by other countries in the Greater Mekong Subregion without proper assessment of health system readiness [48]. Therefore, its fidelity, as measured by the completeness and timeliness of case-based malaria surveillance, has been suboptimal in certain settings because of preexisting health system challenges [16–22]. They included a shortage of health workers to investigate and respond to positive cases and foci; a limited mobile network and internet coverage for timely case notification; the absence of specific and detailed guidelines for reactive case detection; inadequate supplies of diagnostic tools and antimalarial medicines; and the unavailability of funding at health centres to promptly respond to and manage malaria foci [16–22]. All these existing gaps in the health system collectively hinder the national programmes’ efforts to interrupt onwards transmission of malaria and achieve the goal of elimination. In contrast, in Lao PDR, the timeliness of reactive surveillance and response activities improved substantially following health system reform, including the rollout of standardised guidelines, staff training, and the integration of malaria surveillance data into District Health Information Software 2 (DHIS-2)[17]. This example clearly demonstrates how the same strategy could yield different outcomes in countries with varying health system capacities and levels of preparedness.

### Imperfect gauges: why do current tools fail to measure true malaria elimination health system readiness?

Despite the central role of health system readiness assessment and thorough preparation in all three pillars of malaria elimination efforts, no standardised and comprehensive tool currently exists to assess whether health systems are adequately equipped to support and sustain the addition of elimination activities. While several assessment methods and tools exist, their varied scopes and methodologies highlight both strengths and limitations when applied to malaria elimination contexts (Table 2).

**Table 2:** Summary of health system assessment tools and methods, with limitations in the context of malaria elimination

Assessment tool/method	Methodology and assessed domains/key areas	Limitation (s)
Malaria Elimination Audit Tool	National strategy, coordination, policies and advocacy; stratification; diagnosis; case management; surveillance; focus investigations, micro-plans and epidemic response; vector control and entomological surveillance; documents and records for certification of elimination; accelerating strategies; and prevention of re-establishment	Does not adequately assess health workforce planning, access to essential medical products, and health financing
Malaria programme review	Conducted using different methodologies including desk review of published and unpublished papers and reports, stakeholder interviews, focus group discussions, and field visits. Thematic areas to be reviewed vary for each round of review	Lack of a standardised set of tools for each method and variable scopes across the reviews
Service Provision Assessment	Malaria, child health, antenatal care, delivery and newborn care, family planning, nutrition, health system strengthening, water, sanitation and hygiene (WASH), emergency preparedness, gender-based violence, HIV/AIDS, non-communicable diseases, post-abortion care, primary health care, reproductive, cancers, sexually transmitted infections, tuberculosis	Not tailored to the unique demands of malaria elimination programmes, and do not assess readiness for elimination-specific activities such as reactive case detection or foci response
Service Availability and Readiness Assessment	Identification, location and managing authority of health facility; general facility status; basic medical equipment such as X-ray, oxygen, weighing machine; availability of health workforce; availability of general medicines; diagnostic facilities, and specialised services for communicable and non-communicable diseases including malaria; standard and specialised surgery services and blood transfusion	

Among these tools, the Malaria Elimination Audit Tool developed by the WHO offers a structured framework for evaluating the technical and operational domains directly linked to malaria elimination [50]. Its strength lies in its targeted focus on elimination-specific activities, providing a clear lens through which programme managers can assess progress towards zero transmission. However, when assessed against broader WHO health system building blocks, the tool reveals notable gaps: it does not adequately assess critical components such as health workforce planning, access to essential medical products, and health financing. For example, it fails to evaluate whether elimination personnel are adequately deployed and retained to deliver prevention, diagnosis, and treatment services; whether supply chains are robust enough to ensure uninterrupted access to diagnostics and treatments, thereby supporting accelerated efforts towards elimination; or whether financial resources are sustainably allocated to transform malaria surveillance into a key intervention (Table 2). These gaps limit the tool’s utility in informing broader health system readiness assessment for sustaining malaria elimination efforts.

In contrast, the WHO-led periodically conducted malaria programme review offers a more holistic evaluation of national programmes and generates recommendations to enhance operational performance [51]. Its flexibility in selecting and applying different methods—desk reviews, stakeholder interviews, focus group discussions, and field visits—enables context-sensitive insights and fosters stakeholder engagement [52]. However, the lack of a standardised set of tools for each method across the malaria programme reviews presents a significant drawback. The thematic areas of assessment often differ between reviews, leading to inconsistencies in findings and limiting the comparability of results across countries or over time (Table 2) [52, 53]. As a result, while malaria programme reviews can generate rich qualitative insights, their variable scope limits their reliability as tools for longitudinal assessment or cross-country benchmarking to inform evidence-based decision-making, track progress towards elimination goals, and identify best practices that can be adapted across different settings.

Broader health system assessment tools such as the Service Provision Assessment and the Service Availability and Readiness Assessment offer another perspective of health system readiness. These instruments, developed by the Demographic and Health Survey Program and the WHO and the United States Agency for International Development, respectively, are methodologically rigorous and provide standardised data on the service delivery and availability of medical products [54, 55]. Their strength lies in their ability to inform national health planning through robust and generalizable metrics. However, their generalist nature means that they are not tailored to the unique demands of a malaria elimination programme (Table 2). They do not assess readiness for elimination-specific activities outlined in the three pillars of the WHO Global Technical Strategy for Malaria. As a result, while the Service Provision Assessment and the Service Availability and Readiness Assessment can complement malaria programme evaluations, they cannot be used in isolation, as they are not tailored to the specific requirements of elimination strategies.

Taken together, these tools provide only partial insights into health system readiness for malaria elimination and fall short of offering a comprehensive, standardised, and context-sensitive assessment framework. They lack the breadth and depth to assess whether essential health system needs are in place to ensure universal access to malaria services, accelerate efforts towards elimination, and transform malaria surveillance into a key intervention, as outlined in the WHO Global Technical Strategy for Malaria. The absence of a single tool capable of assessing health system readiness for malaria elimination interventions in all three pillars of the WHO Global Technical Strategy for Malaria across six health system building blocks represents a critical gap.

### Advancing health system readiness assessment for malaria elimination and beyond

There is a pressing need for a comprehensive assessment framework that evaluates health system readiness for malaria elimination across all building blocks in a standardised yet adaptable way. Such a framework should identify gaps in governance, financing, workforce, service delivery, information systems, and access to medical products, while reflecting the operational realities of implementing elimination strategies across the three pillars of the WHO Global Technical Strategy for Malaria. Given the context-specific nature of malaria transmission and health system structures, the framework must also be flexible enough to accommodate local epidemiology, institutional arrangements, and population needs.

A promising approach to developing country- or region-specific frameworks is through co-design processes involving key stakeholders, including national malaria programme managers, health system experts, donors, and community representatives. This participatory approach can enhance relevance, acceptance, and ownership [56–58]. However, integrating new practices into existing systems may face barriers such as staff resistance, perceived increases in workload, and workflow disruptions [59]. To mitigate these challenges, targeted advocacy with national programmes and Ministries of Health is essential to strengthen recognition of the importance of health system readiness and routine assessments for malaria elimination.

Furthermore, health system readiness assessment must be recognised as a continuous process rather than a one-time activity. It should be systematically integrated into existing health systems and monitoring and evaluation structures to drive sustained progress in system strengthening. Health policymakers and stakeholders need to prioritise, adopt, and institutionalise readiness assessments as a core component of programme planning and implementation. This approach has clear applicability beyond malaria and should be leveraged more broadly to strengthen health systems across diseases.

## Conclusions

Despite its critical role, assessing health system readiness for malaria elimination remains underrecognised. There is an urgent need for a flexible, comprehensive tool to evaluate readiness across service delivery, surveillance, elimination workforce, products and commodities, programme financing, and leadership and governance while aligning with the WHO Global Technical Strategy for Malaria. Furthermore, health system readiness assessments should be integrated into existing health systems and monitoring and evaluation frameworks to ensure sustained progress in strengthening health systems. Advocacy to key stakeholders is essential to institutionalise these assessments, supported by careful planning and early engagement to enhance ownership and secure institutional support.

Without proper readiness assessment and subsequent strengthening of the health system, the sustainability of national programmes remains uncertain. This perspective underscores the need for comprehensive assessments that consider all aspects of WHO health system building blocks. Such assessments enable policymakers and programme managers to comprehensively evaluate readiness, identify gaps, and sustain efforts towards zero local transmission. Importantly, such an approach strengthens health systems, which are critical for preventing the re-establishment of malaria, maintaining malaria-free status, and achieving the WHO certification of malaria elimination.

## Data Availability

No datasets were generated or analysed during the current study.

## Abbreviations

DHIS-2: District Health Information Software 2
G6PD: Glucose-6-phosphate dehydrogenase
WHO: World Health Organization
